# Supplementary material for: Molecular Regulation of Photosynthetic Carbon Assimilation in Oat Leaves Under Drought Stress
Source: Plants (Basel). 2024 Nov 26;13(23):3317. doi: 10.3390/plants13233317 (PMC11644273; doi:10.3390/plants13233317)
Supplement: Supplementary file 1 [file plants-13-03317-s001.zip › plants-3305870-supplementary.pdf]

Table S1 RNA-seq data Statistics

sample	raw reads	clean reads	clean bases	error rate(%)	Q20%	Q30%	GC pct(%)
baiyan2_0_1	45225428	43424450	6.51G	0.01	97.61	93.61	54.96
baiyan2_0_2	49737798	48128474	7.22G	0.01	98.57	96.2	54.74
baiyan2_0_3	49318102	45406434	6.81G	0.01	97.92	94.38	55.83
baiyan2_6_1	50023244	45629872	6.84G	0.01	98.04	94.72	55.55
baiyan2_6_2	49550302	46990492	7.05G	0.01	97.63	93.68	54.01
baiyan2_6_3	49587298	46283746	6.94G	0.01	97.66	93.8	54.46
baiyan2_12_1	47996336	45353232	6.8G	0.01	98.04	94.65	53.9
baiyan2_12_2	46810902	44359808	6.65G	0.01	98.08	94.78	53.64
baiyan2_12_3	51475228	49107698	7.37G	0.01	97.66	93.77	54.63
